# Supplementary material for: Targeting Fat Oxidation in Mouse Prostate Cancer Decreases Tumor Growth and Stimulates Anti-Cancer Immunity
Source: Int J Mol Sci. 2020 Dec 18;21(24):9660. doi: 10.3390/ijms21249660 (PMC7766808; doi:10.3390/ijms21249660)
Supplement: Supplementary file 1 [file ijms-21-09660-s001.pdf]

Targeting fat oxidation in mouse prostate cancer decreases tumor growth and stimulates anti-cancer immunity

Supplementary Figures and detailed acyl carnitine analysis method.

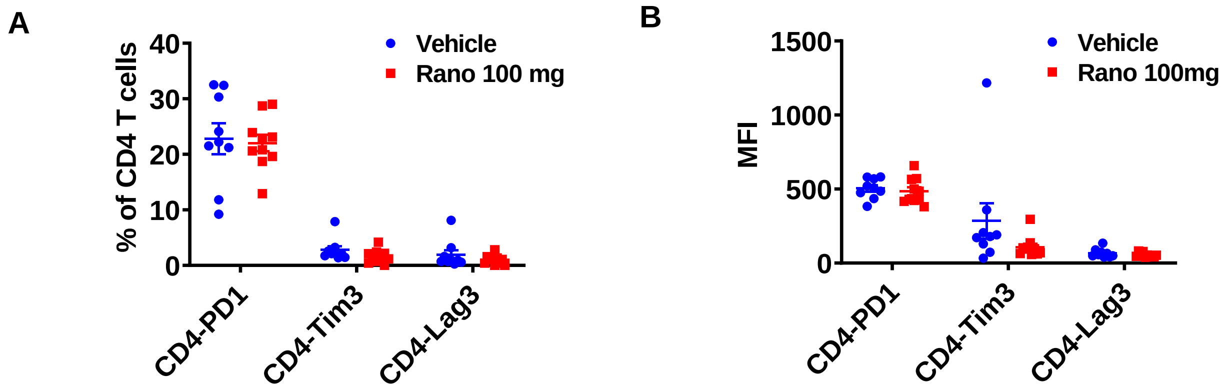

Supplementary Figure 1: Data supplementary to figure 2; CD4 T cell changes in ICP: About 50 mg pieced of tumors of treated mice were collagenase treated and used for flow analysis of ICP surface markers. A-B) Percentage of CD4 T- cells (A) or MFI (B) of PD1, Tim3 and Lag3 ICP markers in exhausted CD4 T-cells from vehicle or treated tumors with 100mg/kg ranolazine (n= 7 mice per group).

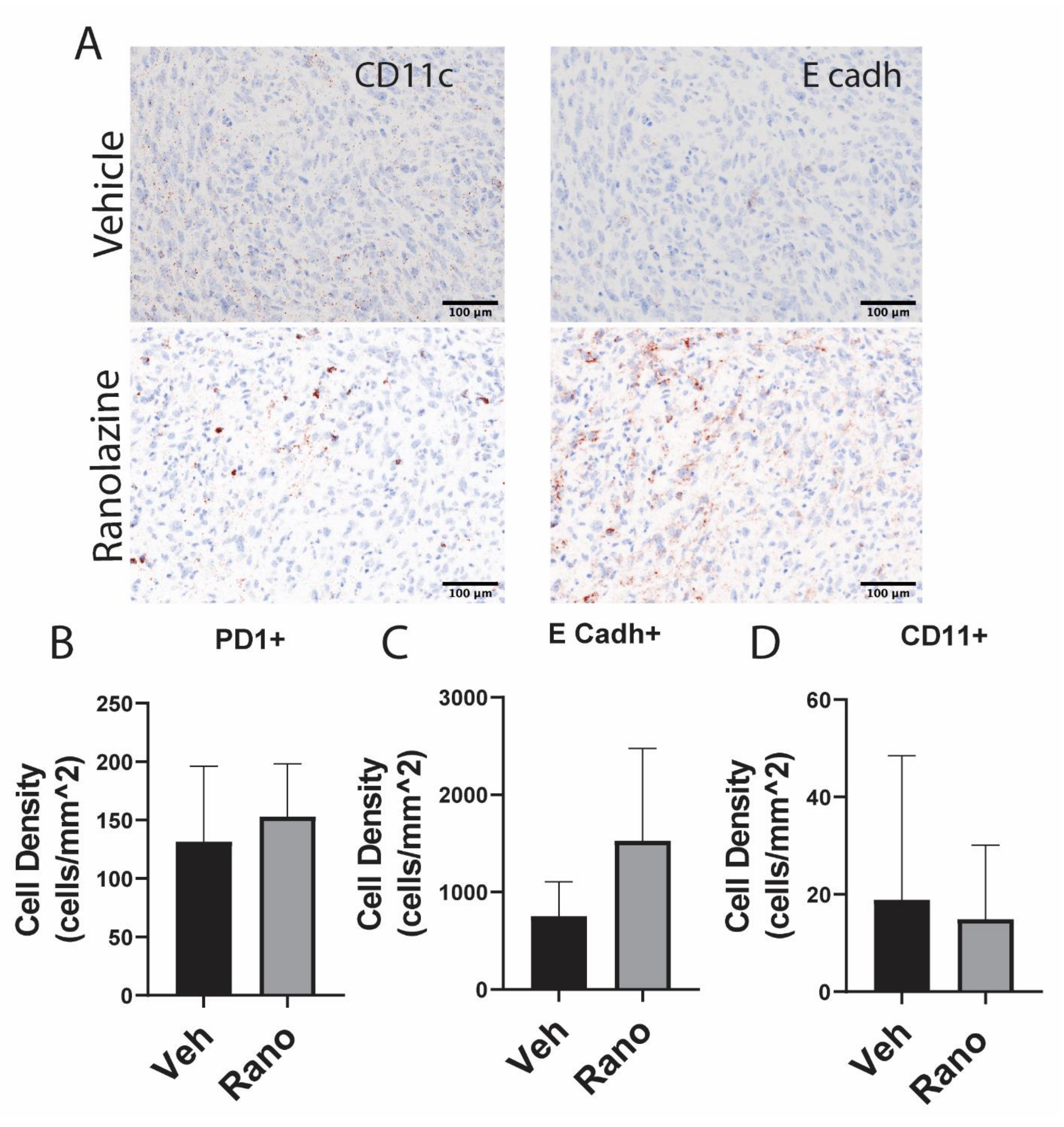

Supplementary Figure 2: Additional quantification of multispectral imaging . Cellular phenotypes were analyzed using inform (Akoya) software and cell density was calculated for PD1+ (A), E-Cadherin+ (B) and CD11+ (C) markers. Comparisons were not significant; n= 7 per condition.

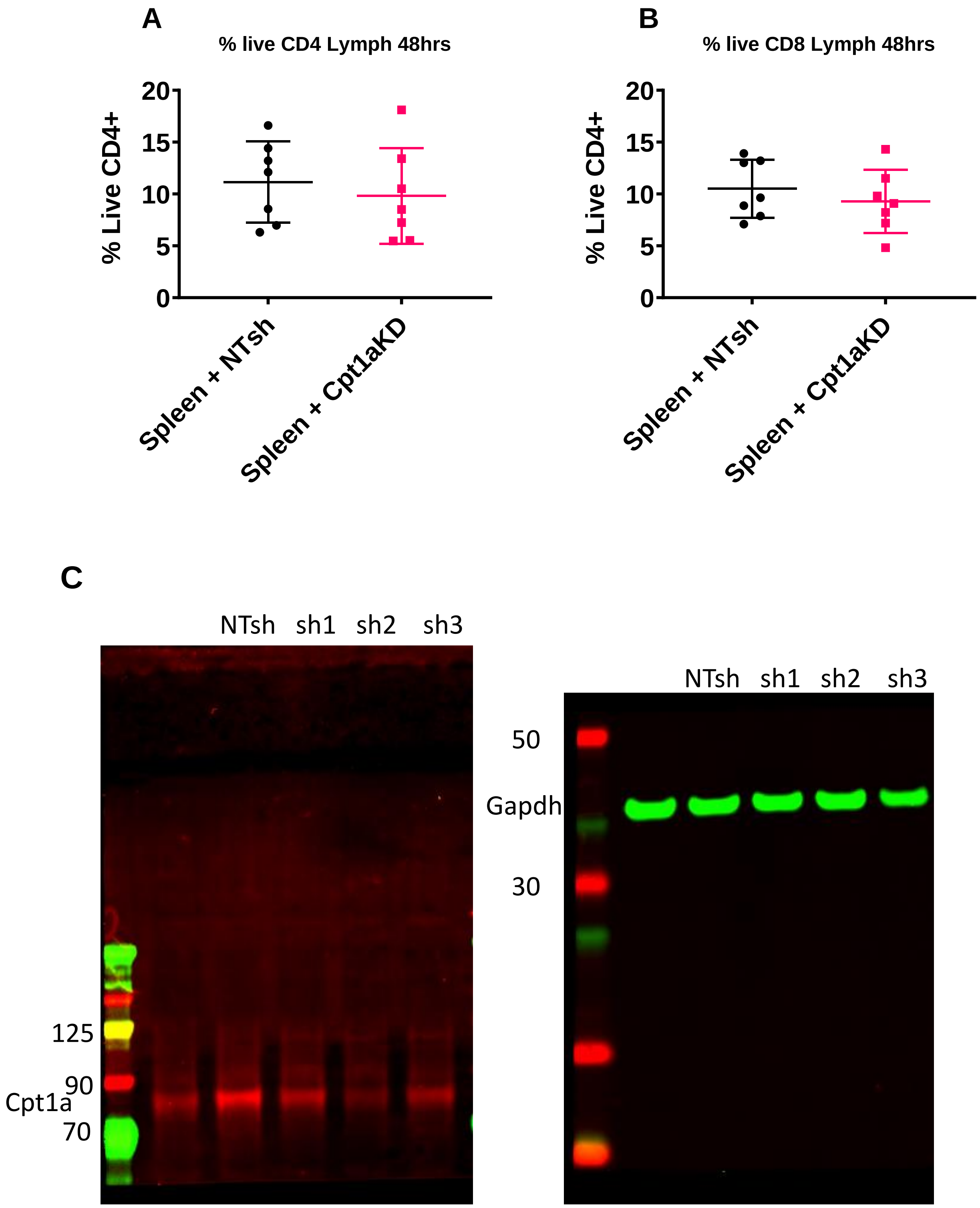

Supplementary Figure 3: Supplementary data to figure 6. Percentage of live CD4 (A) and CD8 (B) lymphocytes after co culturing with Cpt1a-KD TRAMPC1 cells. C) Uncropped LI-COR western blots for Cpt1a and Gapdh in Figure 3A.

Acyl carnitine analysis method:

Cells were homogenized in 500 µL water and an aliquot (20 µL) taken for protein concentration. The homogenized sample was brought up to a total volume of 750 µL with water and methanol (900 µL) was added. After the addition of 8:0-L-carnitine (N-methyl-d3) (2.5 pmol) and 16:0-L-carnitine (N-methyl-d3) (25 pmol) as internal standards, lipid extraction was performed by the addition of methyl-*tert*-butyl ether (3 mL) according to Maytash et al. [1]. The organic phase was dried under a stream of nitrogen gas and resuspended in 400 µL of a mixture of 15% HPLC solvent A (hexane/isopropanol (300/200, v/v) with 10 mM ammonium acetate and 0.056% acetic acid) and 85% solvent B (hexane/isopropanol/water (300/400/84, v/v/v) with 10 mM ammonium acetate and 0.1% acetic acid). Samples were injected into an HPLC system connected to a triple quadrupole mass spectrometer (Sciex 3200, Framingham, MA) and normal phase chromatography was performed using a HILIC column (100x3 mm, Luna HILIC 2.1 µm, Phenomenex). Mass spectrometric analysis was performed in the positive ion mode using multiple-reaction monitoring (MRM) of ten acylcarnitine molecular species and the two deuterated internal standards. The precursor ions monitored were the molecular ions $[M+H]^+$ and the product ion for all acylcarnitine species was at m/z 85 corresponding to loss of fatty acid and trimethylamine [2]. Quantitation was performed using stable isotope dilution with a standard curve for long chain and short chain acylcarnitines and results were normalized to protein content.

1. Matyash, V., et al., *Lipid extraction by methyl-tert-butyl ether for high-throughput lipidomics.* Journal of lipid research, 2008. **49**(5): p. 1137-1146.
2. Kivilompolo, M., et al., *Rapid quantitative analysis of carnitine and acylcarnitines by ultra-high performance–hydrophilic interaction liquid chromatography–tandem mass spectrometry.* Journal of Chromatography A, 2013. **1292**: p. 189-194.
